# Urban-rural differences in health service-related factors associated with male involvement in family planning services in Abia State, Southeastern Nigeria

**DOI:** 10.4314/gmj.v56i3s.12

**Published:** 2022-09

**Authors:** Chidinma I Amuzie, Uche N Nwamoh, Andrew Ukegbu, Chukwuma D Umeokonkwo, Benedict N Azuogu, Ijeoma N Okedo-Alex, Kalu U Kalu, Michael Izuka, Franklin Odini

**Affiliations:** 1 Department of Community Medicine, Federal Medical Centre, Umuahia, Abia State, Nigeria; 2 Nigeria Field Epidemiology and Laboratory Training Program, Abuja, Nigeria; 3 Department of Community Medicine, Alex Ekwueme Federal University Teaching Hospital, Abakaliki, Ebonyi State, Nigeria; 4 African Institute for Health Policy and Health Systems, Ebonyi State University Abakaliki, Ebonyi State, Nigeria

**Keywords:** Male involvement, health service factors, determinants, rural and urban populations, family planning services

## Abstract

**Objective:**

To identify and compare the health service-related factors associated with male involvement in family planning services among the rural and urban areas in Abia State, Nigeria.

**Design:**

A community-based cross-sectional study.

**Setting:**

Twelve communities (six urban and six rural) in Abia State, Nigeria

**Participants:**

Five hundred and eighty-eight (588) men aged 15–59 years and resident in the study area 6 months before the study were recruited.

**Main outcome measure:**

Male involvement in family planning services

**Results:**

The mean ages of the respondents were 41.8±8.0 years and 43.1±8.0 years in the urban and rural areas, respectively. Active male involvement in family planning services was significantly higher in urban areas (62.6%, 95%CI: 56.8%-68.1%) compared to the rural areas (47.6%, 95%CI: 41.5%-53.2%. p<0.001). The predictors of male involvement included gender preference of healthcare workers (aOR=1.75, 95%CI:1.01–3.03) and attitude of the healthcare workers (aOR=2.07, 95%CI:1.17–3.67) among the urban participants, compared to occupational status of the respondents (aOR=2.50, 95% CI: 1.16–5.56) and the availability of male-friendly clinics (aOR=2.27, 95%CI:1.25–4.15) among the rural participants.

**Conclusion:**

Health service-related factors associated with male involvement varied between the urban and rural settings. Stakeholders should target addressing health service-related factors by types of settlement while designing family planning programs targeting men.

**Funding:**

No funding was obtained for this study.

## Introduction

Family planning (FP) programmes have focused primarily on women. However, with a focus on gender equity for optimal health, there is a shift to engage men in supporting and using fp services.^1^ family planning refers to a conscious effort by a couple to limit or space the number of children they want to have through the use of contraceptive methods.

The 2018 Nigeria Demographic Health Survey (NDHS) shows that FP uptake is low, as the overall contraceptive prevalence rate (CPR) among married women was reported to be 17%.^2^ The recommended target, according to the Nigerian government's commitment at the London summit 2012, is to achieve a CPR of 27% by 2020.^3^ In Abia State, rates of 12% and 28.5% of CPR and unmet need for FP were documented, respectively.^4^ Consequently, the Maternal Mortality Ratio (MMR) was 512 deaths/100,000 live births.^2^

Nigeria accounts for roughly one-fifth of all maternal deaths worldwide.^5^ It is the most populous country in Africa and is projected to be the third most populous country globally by 2050.^5^,^6^ The 2018 NDHS report in Nigeria shows that the Total Fertility Rate (TFR) is 5.3 births per 1000 people (expected value is 2.1 births), and it varies with residence (4.5 births urban and 5.9 births rural).^2^

One of the ways to increase FP uptake is by promoting male involvement in FP services. Male involvement in FP refers to all organizational strategies targeted at men as a solitary group with the goal of promoting the use of FP by men or women.^7^ Globally, there is a growing recognition of the benefits of involving men in FP services.^8^ It is believed that engaging men in reproductive health services, especially in patriarchal societies, will enhance the uptake of FP.^7^,^9^,^10^ This is because men are believed to have more access to information and are the major decision-makers in the household.^11^,^12^

Differences in the use of healthcare services between rural and urban areas have been ascribed to many factors, including health service factors in the Nigerian context.^13^–^15^ Some of these factors, as cited in several studies, include gender preference and attitude of healthcare workers, lack of male-friendly services, cost of services, distance to health facilities, and long waiting periods at the health facilities.^16^–^20^ Studies conducted in Africa have reported some factors affecting partner involvement in reproductive health services.^21^–^23^

It has also been reported that disparity in localities affects health service-related factors associated with male involvement in FP.^24^,^25^ Consequently, in Nigeria, evidence from published literature shows that male involvement in FP remains low, and few of these health service factors have been explored for rural-urban disparities in Nigeria.^25^,^26^ There is a need to explore the rural-urban differences of health service-related factors associated with men's involvement in FP services. The findings of this study would guide intervention and policies that could improve male involvement in FP services in all health facilities. The aim of the study was to identify and compare the health service-related factors associated with male involvement in family planning services among urban and rural men in Abia State, Nigeria.

## Methods

### Study design and setting

This community-based cross-sectional study was conducted from September 2019 to February 2020 in 12 communities (6 urban and six rural) in Abia State, South-eastern Nigeria. Abia State had an estimated population of 3,901,620 in 2018, projected from the 2006 national population census with an annual growth rate of 2.7%.^4^ Geopolitically, Abia State is divided into three senatorial zones (Abia North, Abia Central and Abia South) with 17 Local Government Areas (LGAs). Abia State is inhabited mostly by the Igbo ethnic group, who are predominantly Christians.

The State has 517 public primary healthcare centres, 17 public secondary healthcare facilities, and 2 public tertiary healthcare centres. FP services can be accessed at all levels of health facilities in the State, including chemist stores and private health facilities. There are no known existing taboos against FP use in the State. In Nigeria, an urban area is defined as an area with a population size of ≥ 20,000 people, with basic social and physical infrastructure, and so designated through legal or administrative instruments.^27^ Based on the above definitions, the LGAs in Abia State have been categorized into rural and urban in the various senatorial zones (five urban and twelve rural LGAs). Presently, there are 730 Autonomous Communities in Abia State, and each has a traditional ruler known as ‘Eze’.

### Sample size determination

The sample size was estimated using the formula for comparative cross-sectional studies.^28^ This is given as, N = 2(Zα+Zβ)^2^ P(1-P)/ (p_1_-p_2_)^2^ where N is the sample size, Zα and Zβ are the standard normal deviates for the level of significance and power, respectively. P represents the pooled proportion [(p_1_+p_2_)/2] of the FP use in a previous study's rural and urban areas. The p_1_ and p_2_ were the proportions of family planning use among men in rural areas (26.8%) and urban areas (41.2%) in a previous study.^25^ A minimum sample size of 552 (276 for both groups) was determined at a confidence level of 95%, a non-response rate of 10%, a power of 80%, and a design effect of 1.5 was assumed.

### Study population and sampling

The study population included men in a marital or cohabiting relationship with a spouse or partner. This category of men is believed to have had some experiences relating to reproductive health issues in marriage and/or fatherhood. Participants eligible included those aged 15–59 years as adopted from NDHS^29^ and who were living in the study area 6 months before the study. However, those with debilitating illnesses that could interfere with communication were excluded. A total of 588 men were recruited (that is, 294 for the six urban and 294 for the six rural communities) using a multistage sampling technique. In stage one, six LGAs (3 urban and three rural) were selected using the balloting technique. In each senatorial zone, the LGAs were stratified into rural and urban LGAs. One LGA was selected from each stratum in each of the senatorial zones. The selected LGAs included Aba North, Umuahia North, and Ohafia LGAs as the urban areas and Ugwunagbo, Bende, and Ikwuano LGAs as the rural areas for the Abia South, North and Central Senatorial zones, respectively. In stage two, the communities in each of the selected LGAs were enlisted as clusters. These clusters were approximately equal in size. Two clusters were selected in each of the LGAs using balloting.

In each of the clusters, forty-nine respondents were selected. In stage three, the spinning of a pen at the centre of the cluster was done to define the direction of flow to select the households. An eligible respondent was selected in each household visited until the required sample size was attained.

### Data collection tool and procedure

Data were collected using a pre-tested, semi-structured, interviewer-administered questionnaire adapted from previous studies.^26^,^30^ The Igbo-translated version, which was translated back to English to ensure that the original meaning was maintained, was also available for use. The reliability and validity of the questionnaire were assessed using the content and face validity techniques. The Cronbach's alpha index for the English version was 0.71. The questionnaire had three sections. The first section contained socio-demographic variables such as age, religion, denomination and marriage type. The second section contained the health service-related variables such as distance, cost of family planning services, gender preference of healthcare providers, presence of a male-friendly clinic (male-friendly clinics are clinics that are receptive and create an enabling environment for the involvement of men)^18^,^31^, the attitude of healthcare workers, time spent in FP clinics and adequacy of FP services rendered in the FP clinics. The third section focused on the questions to measure the level of male involvement in FP services. Pre-testing was done to assure the appropriateness of the wording and suitability of the questionnaire. The pre-test was conducted in Old Umuahia (Umuahia South LGA) which was not in the study setting, using 60 respondents (10% of the study sample size). Twenty-four (24) research assistants (2 from each of the selected communities) were recruited and trained on the research tools, communication skills, interviewing skills and ethics in research. The research assistants were made up of volunteers recruited from the communities.

### Study variables

The dependent variable was the level of male involvement in FP services. It was created as a composite variable comprising six questions covering respondents' FP practices. The questions included: Are you currently using any family planning method(s)? In the past 3 months, have you ever discussed FP with your spouse/partner? Are you aware of any male FP methods(s)? In the past 3 months have you ever attended a FP clinic? In the past 3 months, have you ever discussed FP with a friend? Would you recommend FP to a friend?

The responses were dichotomized (Yes/No), with a score of “No” = 0 and” Yes” = 1. This gave a maximal score of six (6) and a minimum score of zero (0). A total score of 0 was classified as” non-involvement”, while a score of 1–3 was classified as” passive involvement” and a score of 4–6 was classified as” active involvement”.

For logistic regression, a score of 0-3 was recoded as ‘passive/no involvement’. The independent variables included the socio-demographic and health service-related factors. The variables- cost of transport to clinics, distance to clinics and attitude of health workers were re-coded respectively as follows: “cheap” (not expensive/cheap) and “expensive”, “near” (very near/near), “far” (very far/ far), “short” (normal/short) and “long”, “good” (very friendly/friendly), “poor” (normal/not friendly).

### Data Analysis

Data coding, entry, cleaning, and analysis were done using Epi Info 7.2 software and the IBM SPSS version 26. Univariate analysis was used to compare the distribution of independent variables of respondents by residence. The association between male involvement and the independent variables in FP services was determined using chi-square (χ^2^-test) across both groups of comparison. The variables were dichotomized for ease of data analysis and interpretation. The p-values of less than 0.05 were considered significant. Logistic regression analysis was done to identify the significant predictors of male involvement in FP services for rural and urban areas. Factors that fitted into the regression model, included factors with p values <0.2 at the level of bivariate analysis and those reported from published literature. The level of significance was 5%, adjusted odds ratios and 95% confidence intervals were reported. Appropriate charts and tables were used to display the results.

### Ethical considerations

Approval for this study was obtained from the Ethics and Research Committee of the Federal Medical Center, Umuahia, with reference number FMC/QEH/G.596/ Vol.10/301 and verbal permission was obtained from the ‘Eze’ of each of the 12 communities to be studied. Written informed consent was taken from all the study participants before enrollment into the study. The data were stored on a password-protected computer accessible only to the principal investigator.

## Results

### Socio-demographic characteristics

Out of 600 men approached, 588 agreed to participate, giving a response rate of 98%. Table 1 presents the socio-demographic characteristics of the respondents by locality. The mean age of the respondents was 41.8+ 8.0 years in the urban areas and 43.1+8.0 years in the rural areas. The participants in the urban areas (secondary 47.3%, tertiary 40.5%) were more educated than those in the rural areas (secondary 34.7%, tertiary 35.7% p<0.001).

**Table 1:** Socio-demographic characteristics of Respondents by urban/rural residence (N=588)

Variables	Urban n=294 (%)	Rural n=294 (%)	Total 588 (%)	p-value
**Age group(years)**				
**25 –34**	45 (15.3)	60 (20.4)	105 (17.9)	0.050
**35 – 44**	114(38.8)	127 (43.2)	241 (41.0)	
**≥45**	135 (45.9)	107 (36.4)	242 (41.1)	
**Education Status**				
** No formal education**	10(3.4)	14(4.8)	24 (4.1)	<0.001
**Primary**	26(8.8)	73(24.8)	99 (16.8)	
**Secondary**	139(47.3)	102(34.7)	241 (41.1)	
**Tertiary**	119(40.5)	105(35.7)	224(38.0)	
**Marriage/relationship type**				
**Monogamous**	279(94.9)	269(91.5)	548 (93.2)	0.233
**Polygamous**	9(3.1)	13(4.4)	22 (3.7)	
**Cohabitation**	6(2.0)	12(4.1)	18 (3.1)	
**Current number of living children**				
**None**	4(1.7)	15(5.1)	19 (3.2)	0.071*
**1–2**	64(21.7)	65(22.1)	129 (21.9)	
**3–4**	164(55.8)	160(54.4)	324 (55.1)	
**≥4**	62(21.1)	54(18.4)	116 (19.8)	
**Religion**				
**Christianity**	287(97.6)	286(97.3)	573 (97.4)	0.794
**Traditional**	7(2.4)	8(2.7)	15 (2.6)	
**Denomination** a				
**Catholic**	46(16.0)	85(29.7)	131 (22.9)	<0.001*
**Orthodox**	121(42.2)	67(23.4)	188 (32.8)	
**Pentecostal**	116(40.4)	127(44.4)	243 (42.4)	
**Others**	4(1.4)	7(2.4)	11 (1.9)	
**Duration at the present residence in the community**				
**6 months**	4(1.4)	4(1.4)	8 (1.4)	0.999*
**>6 -12 months**	6(2.0)	7(2.4)	13 (2.2)	
**>12 months- 2years**	22(7.5)	22(7.5)	44 (7.5)	
**>2years**	262(89.1)	261(88.8)	523 (88.9)	
**Occupation Status**				
**Professional**	12(4.1)	16(5.4)	28 (4.8)	0.016
**Trader**	95(32.3)	89(30.3)	184 (31.3)	
**Civil servant**	84(28.6)	62(21.1)	146 (24.8)	
**Skilled manual labour**	28(9.5)	19(6.5)	47 (7.9)	
**Artisan**	35(11.9)	36(12.2)	71 (12.1)	
**Farming**	27(9.2)	54(18.4)	81 (13.8)	
**No occupation**	13(4.4)	18(6.1)	31 (5.3)	
**Educational status of spouse**				0.006
**None**	23(7.8)	11(3.8)	34 (5.9)	
**Primary**	32(10.9)	30(10.2)	62 (10.5)	
**Secondary**	130(44.2)	106(36.1)	236 (40.1)	
**Graduate**	109(37.1)	147(50.0)	256 (43.5)	

P-values < 0.05 are considered significant

* Fisher's exact P

a n=573

A greater proportion of all respondents, 279 (94.9%) for urban and 269 (91.5%) for rural had a monogamous family. The majority of respondents, 283 (96.3%) in urban areas, were living with their spouses, compared to 264 (89.8%) in rural areas. Most urban participants, 164 (55.8%) had about 3–4 children, compared to 160 (54.4%) of rural participants with the same number of children.

**Figure 1:**
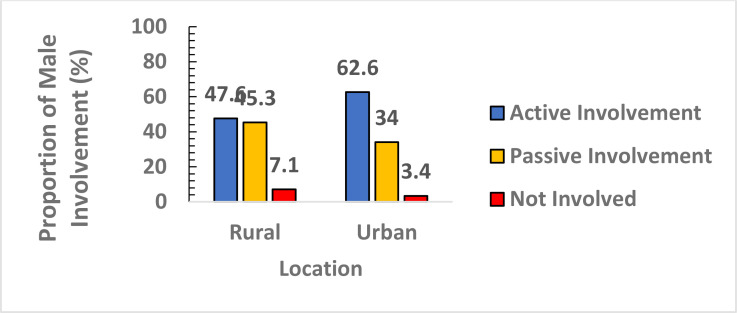
Proportion of Male Involvement in Family Planning Services by residence (N=588)

Figure 1 shows the prevalence of male involvement by residence. Among the urban participants 62.6% (95%CI: 56.8%-68.1%) were actively involved in FP services compared to 47.6% (95%CI: 41.5%-53.2%) of rural participants. Only 10 (3.4%) of the urban participants were not involved in FP services, compared to 21 (7.1%) among the rural participants. This difference was statistically significant (χ^2^ = 14.55, p < 0.001).

### Distribution of health service-related factors among the respondents in urban and rural areas

Table 2 shows the distribution of the health service-related factors among the respondents by place of residence. In the urban areas, more respondents (46.3%) believed that the healthcare workers had good attitude compared to those in the rural areas. (38.4%, p = 0.037).

**Table 2:** Distribution of health service-related factors among the respondents in urban and rural areas (N=588)

Variables	Urban n=294 (%)	Rural n=294 (%)	Total 588 (%)	χ2	p-value
**Cost of transport to FP clinic**					
**Expensive**	37(12.6)	94(32.0)	131 (22.3)	38.31	<0.001
**Not expensive**	126(42.9)	122(41.5)	248 (42.2)		
**Cheap**	131(44.6)	78(26.5)	209 (35.5)		
**Distance of residence to FP clinic**					
**Very far**	7(2.4)	12(4.1)	19 (3.2)	28.78	<0.001*
**Far**	27(9.2)	66(22.5)	93 (15.8)		
**Near**	172(58.5)	149(50.7)	321 (54.6)		
**Very near**	87(29.6)	60(20.4)	147 (25.0)		
**Not aware**	1(0.3)	7(2.4)	8 (1.4)		
**Time spent at FP clinic**					
**Long**	44(15.0)	46(15.7)	90 (15.3)	2.19	0.548
**Normal**	208(70.8)	199(67.7)	407 (69.2)		
**Short**	28(9.5)	38(12.9)	66 (11.2)		
**Not aware**	14(4.8)	11(3.8)	25 (4.3)		
**Attitudes of healthcare worker**					
**Very friendly**	60(20.4)	87(29.6)	147 (25.0)	10.19	0.037
**Friendly**	136(46.3)	113(38.4)	249 (42.3)		
**Normal**	59(20.7)	65(22.1)	124 (21.1)		
**Not friendly**	20(6.8)	19(6.46)	39 (6.6)		
**Not aware**	19(6.5)	10(3.4)	29 (5.0)		
**Is the clinic male-friendly**					
**Yes**	213(72.5)	218(74.2)	431 (73.3)	0.22	0.641
**No**	81(27.6)	76(25.9)	157 (26.7)		
**Gender preference of health worker**					
**Female**	138(46.9)	178(60.5)	316 (53.7)	10.95	0.001
**Male**	156(53.1)	116(39.5)	272 (46.3)		
**Adequate services rendered**					
**Yes**	273(92.9)	273(92.9)	546 (92.9)	3.54	0.188*
**No**	14(4.8)	19(6.5)	33 (5.6)		
**Not aware**	7(2.4)	2(0.7)	9 (1.5)		

P values < 0.05 are considered significant

* Fisher's exact P FP Family Planning

A significantly higher proportion of the respondents in rural areas (31.2%) believed that the cost of transport to the FP clinic was higher than those in urban areas (12.6%, p = 0.001). Similarly, more respondents in the rural areas (4.1%) believed that the clinic was farther away from their homes compared to their urban counterparts (2.4%, p = 0.001). Furthermore, the majority of the rural participants (60.5%), preferred a female healthcare worker compared to urban participants (46.9%, p = 0.001).

### Health service-related factors associated with male involvement in family planning in the urban and rural areas

Table 3 and Table 4 present the results of the bivariate analyses for urban and rural areas, respectively. Only one socio-demographic factor and three health service-related factors attained statistical significance in urban residents. Participants whose spouses' highest educational attainment was secondary education had 43% lower odds of active involvement in FP services. (OR = 0.57, 95% CI; 0.34 – 0.94). However, the odds of active involvement in FP services were 2.38-fold higher among those who perceived the clinic to be near to their residence (OR = 2.38; 95% CI:1.15 – 4.90). Additionally, those who preferred female healthcare workers were more likely to be active (OR = 1.77; 95% CI: 1.09 – 2.86). Lastly, in the urban residence, there was a positive association between the attitude of the healthcare workers and male involvement in FP services (OR = 2.11, 95% CI: 1.24 – 3.60).

**Table 3:** Health service-related factors associated with male involvement in family planning in the urban areas

Variable	Urban n=294(%)					
	Active Involvement	Passive/No Involvement	COR (95%CI)	p-value	aOR (95%CI)	p-value
**Age**						
**<40**	88(47.8)	51(46.4)	1.06 (0.66–1.70)	0.807	1.16 (0.67–1.99)	0.481
**≥40**	96(52.2)	59(53.6)	1			
**Educational Status**						
**Below Tertiary**	106(57.6)	69(62.7)	0.81(0.50–1.31)	0.387	0.93 (0.39–2.22)	0.866
**Occupational Status**						
**Skilled**	63(36.4)	33(30.6)	1.30(0.78–2.17)	0.314	1.39 (0.55–3.57)	0.481
**Unskilled**	110(63.6)	75(69.4)	1			
**Educational status of spouse/partner**						
**Below Tertiary**	107(58.2)	78(70.9)	0.57(0.34–0.94)	0.028	1.75 (0.92–3.31)	0.087
**Tertiary**	77(41.9)	32(29.1)	1			
**Cost of transport to FP clinic**						
**Expensive**	18(9.8)	19(17.2)	0.52(0.30–1.04)	0.061	0.60(0.27–1.34)	0.214
**Cheap**	166(90.2)	91(82.7)	1			
**Distance of residence to FP clinic**						
**Near**	169(91.9)	90(82.6)	2.38(1.15–4.90)	0.017	2.25(0.97–5.19)	0.580
**Far**	15(8.2)	19(17.4)	1			
**Time spent at FP clinic**						
**Long**	28(15.7)	16(15.7)	1.00(0.51–1.96)	0.992		
**Short**	150(84.3)	86(84.3)	1			
**Attitudes of health worker**						
**Good**	134(77.0)	62(61.4)	2.11(1.24–3.60)	0.006	2.07(1.17–3.67)	0.013
**Poor**	40(23.0)	39(38.6)	1			
**Male friendly clinic**						
**Yes**	134(72.8)	79(71.8)	1.05(0.62–1.78)	0.852	-	-
**No**	50(27.2)	31(28.2)	1			
**Gender preference of health worker**						
**Female**	96(52.2)	42(38.2)	1.77(1.09–2.86)	0.020	1.75(1.01–3.03)	0.044
**Male**	88(47.8)	68(61.8)	1			
**Adequate services rendered**						
**Yes**	174(95.6)	99(94.3)	1.32(0.45–3.91)	0.617	-	-
**No**	8(4.4)	6(5.7)	1			

FP Family Planning

* P value <0.05 are considered significant

COR Crude Odds Ratio

aOR Adjusted Odds Ratio

**Table 4:** Health service-related factors associated with male involvement in family planning in the rural areas

Variable	Rural n=294(%)					
	Active Involvement	Passive/No Involvement	COR (95%CI)	p-value	aOR (95%CI)	p-value
**Age**						
**<40**	45(32.4)	71(45.8)	0.57 (0.35–0.91)	0.019	1.40 (0.82–2.40)	0.215
**≥40**	94(67.6)	84(54.2)	1			
**Educational Status**						
**Below Tertiary**	80(57.6)	109(70.3)	0.57(0.35–0.93)	0.023	0.55 (0.24–1.25)	0.155
**Occupational Status**						
**Skilled**	47(34.8)	31(22.0)	1.90(1.11–3.23)	0.018	2.50 (1.16–5.56)	0.019
**Unskilled**	88(65.2)	110(78.0)	1			
**Educational status of** **spouse/partner**						
**Below Tertiary**	67(48.2)	80(51.6)	0.87(0.55–1.38)	0.559	1.02 (0.58–1.81)	0.934
**Tertiary**	72(51.8)	75(48.4)	1			
**Cost of transport to FP clinic**						
**Expensive**	42(30.2)	52(33.6)	0.86(0.52–1.40)	0.541	-	-
**Cheap**	97(69.8)	103(66.5)	1			
**Distance of residence to FP** **clinic**						
**Near**	105(76.1)	104(69.8)	1.38(0.81–2.33)	0.232	-	-
**Far**	33(23.9)	45(30.2)	1			
**Time spent at FP clinic**						
**Long**	17(12.3)	29(20.0)	0.56(0.29–1.08)	0.080	0.70 (0.34–1.45)	0.344
**Short**	121(87.7)	116(80.0)	1			
**Attitudes of health worker**						
**Good**	106(76.8)	94(64.4)	1.83(1.09–3.08)	0.022	1.52 (0.86–2.69)	0.146
**Poor**	32(23.2)	52(35.6)	1			
**Male friendly clinic**						
**Yes**	117(84.2)	101(65.2)	2.84(1.62–4.99)	<0.001	2.53 (1.35–4.76)	0.004
**No**	22(15.8)	54(34.8)	1			
**Gender preference of health** **worker**						
**Female**	90(64.8)	88(56.8)	1.40(0.87–2.24)	0.163	1.20(0.71–2.03)	0.499
**Male**	49(35.3)	67(43.2)	1			
**Adequate services rendered**						
**Yes**	132(95.0)	141(92.2)	1.60(0.61–4.20)	0.331	-	-
**No**	7(5.0)	12(7.8)	1			

FP Family Planning

* P value <0.05 are considered significant

COR Crude Odds Ratio

aOR Adjusted Odds Ratio

For the rural residence, three socio-demographic factors and two health service-related factors were found to be significantly associated with active involvement in FP services. Those less than 40 years had lower odds of active involvement in FP services compared to those older than 40 years (OR = 0.57, 95% CI: 0.35 – 0.91). Similarly, participants with lower educational status were less likely to be actively involved in family planning services compared to those with higher educational status (OR = 0.57, 95% CI: 0.35 – 0.93). However, the odds of active involvement in FP services were 1.9-fold higher among skilled workers compared to unskilled workers (OR = 1.90; 95% CI: 1.11 – 3.23). Additionally, respondents who believed that FP clinic was male-friendly were more likely to be active compared to their counterparts (OR=2.84; 95%CI:1.62 – 4.99). Lastly, in the rural residence, the odds of active involvement was higher among those who believed that the health workers had good attitude compared to those who did not (OR = 1.83, 95% CI:1.09 – 3.08).

The predictors of male involvement in FP in the urban areas included gender preference (aOR:1.75, 95% CI:1.01–3.03) and attitude of the healthcare worker (aOR: 2.07, 95%CI:1.17–3.67). Comparatively, occupational status of respondents (aOR: 2.50, 95% CI:1.16–5.56) and the availability of a male-friendly clinic (aOR: 2.53, 95%CI:1.35–4.76) were the predictors of male involvement in FP in the rural areas.

## Discussion

This study was carried out to determine and compare the health service-related factors among men in urban and rural areas of Abia State. We observed that the predictors of male involvement in FP services were gender preference and the attitude of healthcare workers among the urban participants compared to occupation status of the respondents and the presence of a male-friendly clinic among the rural participants.

The urban respondents had a significantly higher level of active involvement in FP services compared to the rural participants. This is consistent with studies in Southwest and Southeast Nigeria, which observed a low level of involvement in FP in the rural areas.^25^,^26^ A similar study in Gambia reported rural-urban variation in the uptake of FP practices among couples, where the urban couples had a better uptake of FP services.^32^ Additionally, a study conducted in Dhaka, Bangladesh, reported a high level of male involvement in an urban setting.^33^ However, a different finding was observed in a study in Ghana among the Sunyani municipality (urban) which showed that 34.5% of men were involved in FP activities.^30^ Poor rural participation could be attributed to the existence of African patriarchal societies, inadequate male FP methods and prevalent myths and misconceptions about FP use.^10^,^34^ For instance, a study in Togo noted that most of the respondents believed that vasectomy could damage the organs, lead to promiscuity and impair the ability to procreate in the event of the current spouse's demise.^10^. This further promotes the belief that FP should be solely reserved for women.

Among the urban respondents, those who preferred female healthcare workers were likely to be actively involved in FP services. This is in contrast with the finding observed in a study conducted in Southeast, Nigeria^25^ and another study in the Pacific region^35^, which reported more use of FP among people who preferred male healthcare workers. However, finding from a study done in Pakistan was consistent with our study's result.^36^ This finding could be attributed to the fact that people feel women may be more accommodating to clients and skilled in issues that are mostly women-focused, like family planning. In the African setting, female healthcare workers dominate in primary health centers as Community Health Officers (CHOs) and Community Health Extension Workers (CHEWs) compared to their male counterparts, who are rarely seen in the health centers. This norm could also have influenced the perspectives of the respondents.

Respondents who believed that health workers had good attitude were more likely to be involved in FP services in both rural and urban areas. This is consistent with previous studies conducted in Ghana and Tanzania.^30^,^37^,^38^ Furthermore, similar finding was documented in a study done in Pacific region.^35^ Good attitude of a health worker builds the confidence of the client and facilitates the utilization of health services. It also aids patients' satisfaction with health services, leading to more referrals and repeat visits to the clinic. Training sessions on health worker-client relationships should be organized occasionally by hospital managers, especially for workers in FP clinics. Effective interventions such as AIDET (acknowledge, introduce, duration, explanation, thank you) created by the Studer Group^39^ to improve verbal and non-verbal communications within hospitals, should be properly utilized.

Occupation was positively associated with male involvement in FP in the rural areas. Skilled workers had higher odds of active male involvement compared to unskilled workers within the rural locality. This is comparable to prior studies with similar findings,^33^,^40^ where the husband's occupational status had a significantly positive influence on male involvement in FP services. Men's occupation is a key factor for their financial status, which is crucial in deciding family size and uptake of contraceptive methods.^41^ Also, skilled workers are more likely to be exposed to information through networking at workplaces. They are likely to access free healthcare through the National Health Insurance Scheme (NHIS). Additionally, they are more likely to be involved in health promotion to be fit for work. Efforts should be made to improve the occupational status of men by creating more job opportunities for men.

Another predictor was the availability of a male-friendly clinic. This factor was positively associated with male involvement. Comparable findings were noted in other studies.^16^,^35^This was also consistent with the findings in a qualitative study conducted in Ghana**.**^37^ Men are reluctant to visit the clinic because it is considered a “female” environment. Long waiting times in clinics are likely to deter men who need to return to work quickly.^42^ Physical layout of the clinics is also known to contribute, as there may be no separate rooms for private consultations. There is a need for stakeholders to provide the male-friendly enabling environment for the delivery of FP services. This should include having a stigma-free setting that will make men comfortable, providing information that will also address men's needs and developing information education counseling (IEC) materials that target men to be involved in FP services.

There were limitations to the findings of this study. The first was social desirability bias and the use of self-reported data from the respondents. People are generally sensitive to reproductive health discussions, which could have led to socially desirable answers. Secondly, the literature review conducted thus far showed that there was no single established index for assessing male involvement in FP services. This could account for the differences between this study and other studies whose collection of data did not employ the same data collection tool. Lastly, there was a potential for recall bias. To mitigate these limitations, the research assistants were well trained on the data collection process; an extensive literature review to aid the operationalization of the outcome variable was done and a short recall period of 3 months was used.

Despite these limitations, the study provides insight into urban-rural differences in health service-related factors influencing male involvement in FP services.

The results of this study align with the International Conference on Population and Development (ICPD) objectives as adopted in reproductive health policy. Furthermore, it was a community-based study, increasing the generalizability of the findings.

## Conclusion

This study showed that about two thirds of urban participants were actively involved, compared to less than half of rural participants. The predictors of male involvement in FP services were gender preference and the attitude of healthcare workers among the urban participants compared to occupation status of the respondents and the presence of a male-friendly clinic among the rural participants. Therefore, there is a need for policymakers and stakeholders to consider these urban-rural differences in health service-related factors when designing family planning interventions targeting men in Abia State, Nigeria.
